# Editorial: A Broader View for Plant EvoDevo: Novel Approaches for Diverse Model Systems

**DOI:** 10.3389/fpls.2017.00061

**Published:** 2017-01-26

**Authors:** Verónica S. Di Stilio, Rainer Melzer, Jocelyn C. Hall

**Affiliations:** ^1^Department of Biology, University of WashingtonSeattle, WA, USA; ^2^School of Biology and Environmental Science, University College DublinDublin, Ireland; ^3^Department of Biological Sciences, University of AlbertaEdmonton, AB, Canada

**Keywords:** evolution of development, emerging model systems, land plants, plant morphology, evolutionary transitions, developmental genetics, phylogenetics, molecular toolkit

For many years, a main focus of plant evolutionary developmental biology was studying the expression and phylogenetic history of genes implicated in developmental pathways. This approach has been enormously successful in identifying potentially conserved gene regulatory circuits that underlie major pattern formation processes in plants. Importantly, hypotheses were often generated on how changes in these gene regulatory-circuits led to the evolution of different plant forms. However, for quite some time, experimental testing of many of these hypotheses proved difficult, simply because the adequate molecular biology toolkit was not available across many plant lineages. This situation has changed dramatically in recent years. The advent of next generation sequencing considerably facilitated sequencing genomes and transcriptomes of plants throughout the phylogeny. Virus-induced gene silencing and the establishment of transformation methods for non-model plants enabled direct testing of gene functions on a wide phylogenetic spectrum, and elaborate biophysical techniques are increasingly applied to analyze changes in protein function during evolution. Furthermore, bioinformatics as well as systems biology are used to integrate the available data into a more coherent understanding of a fundamental question in plant evolution: What are the molecular underpinnings for the origin of different plant forms? Among the many facets this question touches are the transition to land, the emergence of vascular plants, the origin of the seed and the origin and diversification of floral form. In this research topic we highlight emerging model systems across the land plant phylogeny as well as exciting current approaches, including genomics, biophysics, gene networks and transgenesis of plants from diverse lineages. We aim to bring to the forefront the most salient and original plant systems and approaches within an inclusive phylogenetic context that encompasses representatives of the major lineages of land plants.

Among the novel experimental approaches that can be applied in a variety of systems, three articles offer perspectives on methodologies for the study of the diversification of form and the divergence of species. In interspecies gene transfer (IGT), Nikolov and Tsiantis describe how candidate genes from a donor species are added to the wildtype genome of a recipient species to test their causality in the divergence of the two species. In evolutionary transgenomics, Correa and Baum describe the transfer of whole genomic fragments between species to identify novel genes of large effect without prior commitment to candidate genes. Silva et al. discuss the importance of biophysical studies for understanding morphological evolution. They show that small changes in the amino acid sequence of floral developmental regulators can lead to drastically altered protein–protein interaction patterns that may in turn have contributed to the evolution of the flower. This result illustrates that a more integrated approach—using genetics, biophysics and phylogenetics—is necessary to understand the evolution of development.

Several articles present emerging model systems for the study of plant evo–devo, from shoot evolution to changes in inflorescence and floral traits. Plackett et al. highlight the use of the emerging model fern *Ceratopteris richardii*, in the sister lineage to seed plants, for the study of the evolution and development of shoots, and of the genetic regulation of shoot apical meristems. Among the non-flowering seed plants, conifers represent a close extant relative of flowering plants, making them especially interesting from an evolutionary developmental perspective. Uddenberg et al. highlight the importance of studying conifers and suggest that next generation sequencing and improved transformation protocols will make them more accessible to evo–devo studies. Within angiosperms, Vandenbussche et al. argue that new “supermodels” are required to comprehensively study the evolution of gene function that would ideally be as amenable to genetic analyses as *Arabidopsis*. The authors suggest that petunia could be one of those supermodels and provide an extensive overview of the genetic resources available for this system. Cronk et al. describe the evolution of catkin inflorescences in Salicaceae (poplars and willow) illustrating how the morphological richness of the Salicaceae coupled with the rapidly expanding genomic resources make this, of all woody plant families, particularly promising for genome-enabled evolutionary developmental biology. Landis et al. present *Saltugilia* (Polemoniaceae) as a model for the study of flower size (corolla tube length), a trait central to pollination syndrome. They find two independent evolutionary transitions to long corollas, and a correlation of long corollas with an increase in jigsaw cell size and number and with the up regulation of genes associated with cell wall formation and organization. Morioka et al. examine floral diversity in Zingiberales, where members of Cannaceae have a laminar style that plays an important role in pollination interactions. Expression and evolution of genes involved in adaxial/abaxial polarity reveal a complex evolutionary history and suggest that loss of expression lead to this novel feature in *Canna*. Pabón-Mora et al. investigate the genetic basis of the highly derived and fused morphology of *Aristolochia fimbriata*. Developmental and comparative gene expression data support that the fused perianth is derived from sepals, not petals. Their data also reveal that A-class genes in the classic ABCE model do not contribute to perianth identity in this system. This finding provided further evidence that *Arabidopsis* A-class orthologs rarely contribute to perianth identity in other taxa.

Three articles present evidence for opposing forces in the evolution of developmental mechanisms: conservation and divergence of gene and protein function. On the one hand, Hirakawa and Bowman show evidence for the conservation of protein function of the CLE family peptide hormone Tracheary element Differentiation Inhibitory Factor (TDIF) in regulating procambial cell fate, an important aspect in the evolutionary transition to vascular plants. The study performed evolutionary and functional comparative analyses, using protein assays, among representatives of major lineages in vascular plants and concluded that TDIF was integrated into shoot xylem differentiation in the euphyllophyte lineage (ferns and seed plants), after the split from lycophytes. Vialette-Guiraud et al. also present evidence for a conserved gene regulatory circuit, this time during flower development: They show that a genetic module consisting of microRNA164 and NAM transcription factors is responsible for the fusion of carpel margins in eurosids. The authors further suggest that the same gene regulatory circuit could have contributed to the emergence of the closed carpel very early during angiosperm evolution, and might thus have been involved in the origin of one of the most important evolutionary novelties in angiosperms. On the other hand, Yu et al. show that the exon-intron structure of genes is labile and influences the evolution of flower development gene lineages, frequently following gene duplication and speciation events. Their work suggests, for example, that an unstable gene structure in the *AGL6* lineage may have contributed to its functional diversification in the flowering plants and to its divergence from the *SEP* lineage, which went on to become the major mediator of angiosperm floral quartets.

We hope that the publication of this research topic will promote renewed interest in the need for a larger inventory of model systems and approaches to facilitate an increasingly broader and more meaningful view of the evolution of plant development.

## Author contributions

All authors listed, have made substantial, direct and intellectual contribution to the work, and approved it for publication.

## Funding

VD was funded by NSF-IOS 1121669 and RM acknowledges support from University College Dublin.

### Conflict of interest statement

The authors declare that the research was conducted in the absence of any commercial or financial relationships that could be construed as a potential conflict of interest.

